# SGLT2 Inhibitor Canagliflozin Alleviates High Glucose-Induced Inflammatory Toxicity in BV-2 Microglia

**DOI:** 10.3390/biomedicines12010036

**Published:** 2023-12-22

**Authors:** Ching-Tien Lee, Kun-Der Lin, Cheng-Fang Hsieh, Jiz-Yuh Wang

**Affiliations:** 1 Department of Medical and Healthcare Business, Hsin-Sheng College of Medical Care and Management, Taoyuan 32544, Taiwan; chingtien1213@gmail.com; 2 The Lin’s Clinic, Kaohsiung 80778, Taiwan; berg.kmu@gmail.com; 3 Division of Geriatrics and Gerontology, Department of Internal Medicine, Kaohsiung Medical University Hospital, Kaohsiung 80756, Taiwan; skywalker_hsieh@hotmail.com; 4 Graduate Institute of Medicine, College of Medicine, Kaohsiung Medical University, Kaohsiung 80708, Taiwan; 5 Neuroscience Research Center, Kaohsiung Medical University, Kaohsiung 80708, Taiwan; 6 Research Center for Precision Environmental Medicine, Kaohsiung Medical University, Kaohsiung 80708, Taiwan; 7 Department of Medical Research, Kaohsiung Medical University Hospital, Kaohsiung 80756, Taiwan

**Keywords:** SGLT2 inhibitor, canagliflozin, microglia, inflammation, hyperglycemia, diabetes mellitus, neurodegeneration

## Abstract

Patients with diabetes mellitus can experience hyperglycemia, which affects brain function and produces cognitive impairment or neurodegeneration. Neuroinflammation is an important cause of cognitive dysfunction. Sodium-glucose cotransporter 2 (SGLT2) inhibitors are antihyperglycemic agents that reportedly possess anti-inflammatory properties and may produce beneficial cognitive effects. We hypothesized that SGLT2 inhibitors alleviate hyperglycemia-related inflammation in brain immune cells. Cultured BV-2 microglia were exposed to high glucose (HG) in the absence or presence of SGLT2 inhibitors including canagliflozin (Cana), dapagliflozin (Dapa), empagliflozin (Empa), and ertugliflozin (Ertu). Afterward, we evaluated the cytotoxic and inflammatory responses by specific biochemical assays. Treatments with non-toxic Cana or Dapa, but not Empa or Ertu, inhibited proliferation without cell death. Only Cana rescued BV-2 microglia from HG-induced cytotoxicity, including apoptosis or autophagic degradation. None of SGLT2 inhibitors affected the HG-stimulated induction of stress proteins HO-1 and HSP70. Also, compared to the other three SGLT2 inhibitors, Cana was better at inhibiting HG-induced oxidative/inflammatory stress, as evidenced by its ability to repress proinflammatory factors (e.g., oxygen free radicals, iNOS, NLRP3, IL-1β, and TNF-α) other than COX-2. Cana’s action to alleviate HG insults was mediated not by altering SGLT2 protein expression, but by reducing HG-stimulated signaling activities of NFκB, JNK, p38, and PI3K/Akt pathways. Particularly, Cana imitated the effects of NFκB inhibitor on HG-induced iNOS and COX-2. Of the four SGLT2 inhibitors, Cana provided BV-2 microglia with the best protection against HG-induced inflammatory toxicity. Thus, Cana may help to reduce innate neuroimmune damage caused by hyperglycemia.

## 1. Introduction

Type 2 diabetes mellitus (T2DM) is an age-related chronic disease characterized by cellular insulin resistance, metabolic abnormalities, and chronic inflammation. T2DM accelerates aging in most organ systems, leading to premature morbidity and mortality [1]. The brain-related effects of T2DM are well-recognized. Noteworthily, T2DM is a major risk factor for cognitive decline and Alzheimer’s disease (AD), the most common form of dementia [2,3]. Further, verifying T2DM’s impact on AD has prompted active exploration of the mechanisms which underlie and link the two diseases. Evidence from epidemiological and clinical observations, animal studies, and in vitro experiments has established a strong correlation between T2DM and AD pathophysiology [4]. For instance, T2DM is associated with AD symptoms, including dementia, cognitive deficits, hippocampal and cortical cell damage, deposition of amyloid plaques, and fibrillary tangles [5]. The increasing prevalence of diabetic dementia is an area of great public concern that must be solved. 

Studies have identified several overlapping mechanisms related to neurodegeneration in patients with T2DM and dementia, including oxidative stress, mitochondrial dysfunction, and inflammation [2]. Advanced glycation end products generated by hyperglycemia and their receptor have been reported to be a key link between T2DM and AD [6]. Although the brain is an immune-privileged organ, crosstalk between central and peripheral inflammation sites has been reported. The impairment of blood brain barrier (BBB) integrity by hyperglycemia is apparent in diabetics and leads to the infiltration of circulating immune cells or factors into the brain, potentially exacerbating central inflammation [7]. The consequent, chronic, and low-grade inflammation has been proven to promote neuronal loss [8]. Microglia-mediated neuroinflammation has emerged as a leading cause of cognitive dysfunction, providing a central pathogenic mechanism for hyperglycemic neuropathy [9,10]. Microglia are highly susceptible to pathological brain changes. Upon activation, they produce excessive detrimental substances such as nitric oxide (NO), oxygen free radicals, and proinflammatory cytokines (e.g., interleukin (IL)-1β and tumor necrosis factor (TNF)-α), to initiate and promote pro-inflammatory responses, leading to neuronal death and even injuring the microglia themselves [11]. If BBB dysfunction occurs, acute peripheral hyperglycemia readily stimulates microglial activity, followed by chronic self-degradation, i.e., apoptosis or autophagy, under hyperactivated microglia-driven inflammatory toxicity. Eliminating the contributions of microglia to inflammation may help to prevent T2DM-related brain degeneration.

New antidiabetic agents, sodium-glucose cotransporter 2 (SGLT2) inhibitors, have been approved for T2DM treatment [12]. The SGLT2 inhibitors including canagliflozin (Cana), dapagliflozin (Dapa), empagliflozin (Empa), and ertugliflozin (Ertu) have received substantial attention because of the unique glucose-lowering ability in the kidney and cardiovascular protection against inflammation [13,14]. SGLTs naturally exist within the mammalian brain [15,16], while SGLT inhibitors are lipid-soluble and readily cross the BBB [12]. Notably, SGLT2 inhibitors have exerted glycemic control effects and improved cognitive function in diabetic patients and diseased animal models (e.g., db/db or high fat diet (HFD)-fed mice) [17,18,19,20,21,22]. The mechanisms underlying the beneficial effects of SGLT2 inhibitors likely involve anti-inflammation, reduction of cerebral oxidative stress and insulin resistance, and improvement of peripheral glucose metabolism [20,21,22,23]. Despite these revelations, the effects of SGLT2 inhibitors on microglia-mediated neuroinflammation induced by hyperglycemia remains unclear. 

This study investigated whether SGLT2 inhibitors alleviate hyperglycemia-related gliotoxicity and inflammation using an in vitro cell culture model of murine BV-2 microglia. To mimic acute hyperglycemia, cell medium was rapidly shifted from normal to high glucose (HG). We measured several biochemical parameters to assess cytotoxicity and inflammation after HG treatment with or without the presence of SGLT2 inhibitors. The aim was to perform a parallel comparison of the efficacy of four SGLT2 inhibitors, indicating which one is superior to the other three in inhibiting HG-induced microglial toxicity and inflammatory response. Among the SGLT2 inhibitors tested at the same concentration levels, Cana stood out as a medicine candidate due to its excellent efficacy.

## 2. Materials and Methods

### 2.1. Chemicals, Kits and Reagents

Unless stated otherwise, all chemicals were from Sigma-Aldrich (Saint Louis, MO, USA). The kits used for enzyme-linked immunosorbent assay (ELISA) and subcellular fractionation were purchased from R & D Systems (Minneapolis, MN, USA) and BioVision Inc (Mountain View, CA, USA), respectively. Bromodeoxyuridine (BrdU) was purchased from AAT Bioquest (Sunnydale, CA, USA). 2′,7′-Dichlorodihydrofluorescein diacetate (H_2_DCFDA) was purchased from Molecular Probes (Invitrogen, Carlsbad, CA, USA). Chemical agents, including mitogen-activated protein kinase (MAPK) inhibitor PD98059, c-Jun N-terminal kinase (JNK) inhibitor SP600125, p38 MAPK inhibitor SB203580, and phosphatidylinositol 3-kinase (PI3K) inhibitor LY294002, were purchased from Selleckchem (Houston, TX, USA). Nuclear factor-κB (NFκB) activation inhibitor caffeic acid phenylethyl ester (CAPE) and four SGLT2 inhibitors, Cana, Dapa, Empa, and Ertu, were purchased from Cayman Chemical (Ann Arbor, MI, USA). All cell culture reagents were obtained from Gibco-BRL/Invitrogen (Carlsbad, CA, USA). Details related to the antibodies used are provided in the Supplementary Materials.

### 2.2. Cell Cultures and Treatments

The mouse microglial cell line BV-2 (Cat.# ABC-TC212S) was obtained from AcceGen (Fairfield, NJ, USA) and permanently cultured in a growth medium consisting of low-glucose Dulbecco’s modified Eagle’s medium (DMEM; 5.5 mM glucose) containing 10% heat-inactivated fetal bovine serum, 2 mM L-glutamine, 1% penicillin/streptomycin and 1 mM sodium pyruvate at 37 °C in a humidified incubator with 95% air/5% CO2. Fifteen cell passages between thawing and usage for experiments were performed. Unless otherwise indicated, cells were seeded in 6-well or 24-well plates at a density of 1.5 × 10^5^ cells/mL for a series of experiments. 

All the treatments were performed under serum-free conditions. To induce HG stimulation, cell media with normal glucose (NG; 5.5 mM) was replaced by fresh media containing different HG levels (25, 50, 75, 100, or 125 mM, referred to as NG-to-HG) for the indicated times (see Figure legends for details). In terms of applying SGLT2 inhibitors, NG-cultured cells were initially exposed to SLGT2 inhibitors (10 or 20 μM) for 30 min. Subsequently, they underwent varying durations (i.e., 30 min, 6, 12, 24, or 48 h) of HG treatment (50 or 100 mM) while continuously exposed to SLGT2 inhibitors. Dimethyl sulfoxide [DMSO; 0.1% (*v*/*v*)] was used as vehicle for SGLT2 inhibitors. The control group refers to the untreated cultures under NG condition. A constant NG condition had its cell media replaced without glucose concentration alteration. Cells were also treated with various high mannitol concentrations (5.5 mM glucose plus mannitol at 19.5, 44.5, 69.5, 94.5, or 119.5 mM) to act as a purely hyperosmolar control.

### 2.3. Cell Viability Assay

The cells’ 3-(4,5-dimethylthianol-2-yl)-2,5 diphenyl tetrazolium bromide (MTT) reduction ability was measured to assess cell viability. The stock MTT solution prepared in phosphate-buffered saline (PBS) was added to reach a final concentration of 0.5 mg/mL, then cells were incubated at 37 °C for 2 h. Theoretically, viable cells with active metabolisms convert MTT into purple formazan crystals. After removing MTT-containing media and dissolving the crystals with DMSO, the quantity of formazan products was measured by recording changes in absorbance at 570 nm against a 620 nm reference wavelength (ΔOD; OD: optical density) using a Multiskan microplate reader (Thermo Fisher Scientific, Waltham, MA, USA). The percent of surviving cells was calculated as (viable cells) % = (ΔOD of treated sample/ΔOD of vehicle-treated or untreated control) × 100. The vehicle-treated or untreated control well was assigned a value of 100% cell survival.

### 2.4. Measurement of Nitrite Accumulation

The stable conversion product of NO, nitrite (NO_2_^−^), was measured to assess the NO concentrations in cultures. An aliquot of culture supernatant was added to an equivalent volume of Griess reagent (a mixture of 1% sulfanilamide plus 0.1% N-(1-Naphthyl) ethylenediamine dihydrochloride in 5% H_3_PO_4_) in 96-well plates and incubated for 10 min at room temperature. Next, nitrite levels were measured colorimetrically using a Multiskan microplate photometer (Thermo Fisher Scientific) at 540 nm. Finally, the concentration value was calculated by comparing to the standard curve made by sodium nitrite.

### 2.5. Quantifications of TNF-α and IL-1β by ELISA

Media fractions of cultured cells were used to evaluate secreted cytokine levels. The standard ELISA protocol based on the quantitative sandwich enzyme immune-assay technique was used to quantify the released TNF-α and IL-1β levels. The commercially available ELISA kits were employed according to the manufacturer’s instructions. The serial dilutions of recombinant mouse TNF-α and IL-1β served as the standards for constructing the standard curves. Finally, the resulting curves were used to calculate concentration values for each sample.

### 2.6. Intracellular Reactive Oxygen Species (ROS) Measurement

Intracellular ROS production was measured as the fluorescence intensity of 2′,7′-dichlorofluorescein, an oxidation product of H_2_DCFDA. After treatment, the cultures were incubated with 10 μM of the cell-permeant H_2_DCFDA for 30 min at 37 °C and the fluorescence intensity of 2′,7′-dichlorofluorescein was detected at an excitation wavelength of 485 nm and an emission wavelength of 528 nm on an FLx800 fluorescence microplate reader (BioTek, Winooski, VT, USA). The groups’ values (fluorescence readings) are presented as relative fluorescence intensities calculated via comparison with control group.

### 2.7. Immunofluorescence (IF) Staining

Initially, cells (5 × 10^4^ cells/mL) were cultured on sterile coverslips that had been plated in 6-well plates. After completing the indicated treatments, cells were sequentially rinsed with PBS, fixed in methanol, permeabilized with 0.3% Triton X-100, and blocked with 0.5% BSA at room temperature. Next, cells were incubated overnight at 4 °C with a rabbit anti-NFκB p65 antibody (#8242; 1:200), followed by Alexa Fluor 488-conjugated secondary antibody (111-545-003; 1:400) for 2 h at room temperature in the dark. Then, cells were stained with 1 μg/mL of 4′,6-diamidin-2-phenylindol (DAPI) in PBS for 10 min at room temperature. Finally, cover slides were mounted and analyzed under an Axioplan2 fluorescence microscope (Zeiss, Oberkochen, Germany).

### 2.8. Bromodeoxyuridine (BrdU) Labeling for In Vitro Cell Proliferation Assay

In vitro BrdU labeling combined with IF staining was used to assess cell proliferation. Initially, cells were seeded as for IF staining. BrdU (10 μM) was added to the cultures 2 h before cells were harvested at 24 h. Next, cells were washed, fixed, permeabilized, DNA denatured, incubated with BrdU antibody, and more. For further details, see Supplementary Materials.

### 2.9. Western Blotting and Subcellular Fractionation

Protein levels of interest were assessed according to our established protocol in combination with commercially available kits. Briefly, cells were lysed with RIPA buffer. After protein quantification, the samples (30 μg) were separated by electrophoresis and transferred onto PVDF membrane. Then the membrane was incubated with primary antibody against the target protein, followed by incubation with corresponding secondary antibody. Finally, the signals were visualized with enhanced chemiluminescence method. Please see Supplementary Materials for detailed procedures.

### 2.10. Statistical Analysis

Data are expressed as mean ± SEM. Statistical analyses and bar graph plotting were respectively performed using the SigmaStat version 4.0 and SigmaPlot version 10.0 software from Jandel Scientific (San Diego, CA, USA). The Kolmogorov–Smirnov test was used to test data for normality, while the Levene Median test was used for testing equal variance assumption. Statistical differences in all parameters were analyzed using a one-way ANOVA followed by either Dunnett’s test for comparisons versus a control group or by a Bonferroni *t*-test for post-hoc multiple pairwise comparisons. The *n*-value represents the number of repetitions per group. For all comparisons, *p* < 0.05 was considered statistically significant. 

## 3. Results

### 3.1. Cana and Dapa Attenuate BV-2 Microglial Proliferation

**Figure 1:**
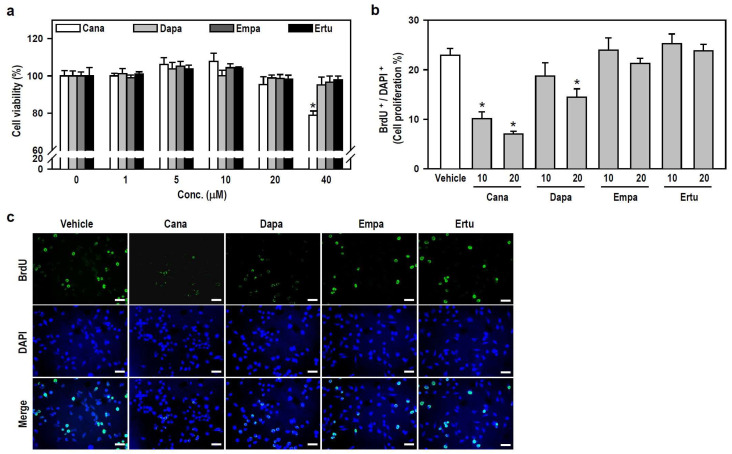
Both SGLT2 inhibitors, Cana and Dapa, decrease BV-2 cell proliferation at non-toxic concentrations. (**a**) Cell viability was assessed using the MTT assay after treating the cultures with various concentrations (0–40 μM) of Cana, Dapa, Empa, and Ertu for 24 h. The data were presented as a histogram and are expressed as mean ± SEM (*n* = 5, in triplicate). * *p* < 0.05 vs. vehicle-treated control group (0 μM; assigned a value of 100%). (**b**,**c**) Cultures were treated with either 10 or 20 μM of each inhibitor for 24 h. BrdU labeling for the in vitro cell proliferation assay was performed to recognize proliferating cells. Representative photomicrographs of BrdU-positive cells (green) and DAPI-labeled nuclei (blue) were shown. Scale bar = 50 μm. Quantitative results were presented using a histogram. Values are expressed as mean ± SEM of one representative experiment, two other repetitions showed consistent results (Figure S1). * *p* < 0.05 vs. vehicle-treated control group.

To characterize the action of SGLT2 inhibitors on microglial cell activity, we assessed the viability and growth of cultured BV-2 cells. Within the applied concentration ranges, only 40 μM Cana evidently decreased cell viability (Figure 1a), indicating that SGLT2 inhibitors, at concentrations ≤20 μM, are nontoxic to BV-2 microglia. Accordingly, we chose two nontoxic concentrations, 10 and 20 μM, to execute a series of follow-up experiments involving parallel comparisons of four SGLT2 inhibitors.

We subsequently examined whether nontoxic SGLT2 inhibitors affect the growth of BV-2 microglia. Compared with the vehicle-treated control, both Cana (10 or 20 μM) and Dapa (20 μM), rather than Empa and Ertu, significantly suppressed cell proliferation, as determined by the ratio of BrdU-positive cells to 4′,6-diamidin-2-phenylindol (DAPI)-labeled nuclei (Figure 1b,c and Figure S1). These results suggest that Cana shows excellent anti-proliferative effect on microglial cells.

### 3.2. HG Induces p53-Associated Gliotoxicity Leading to Apoptosis and Autophagic Degradation in BV-2 Microglia

**Figure 2:**
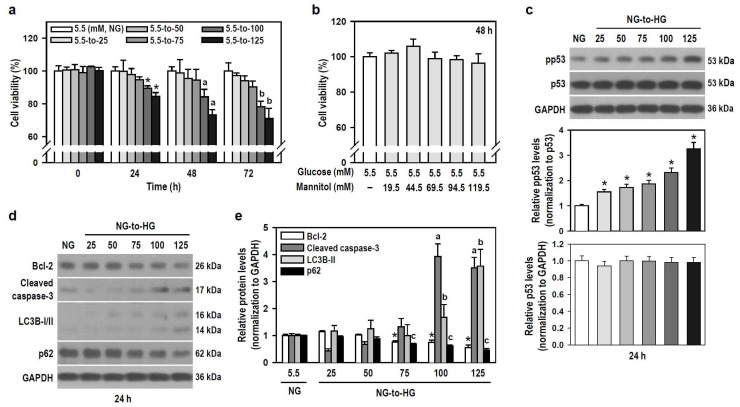
HG reduces the viability of BV-2 cells in a time- and concentration-dependent manner through apoptosis and autophagy. (**a**,**b**) Cultures were treated with glucose concentrations ranging from 5.5 to 125 mM for different and independent durations, as indicated. Also, cultures were exposed to increasing concentrations of a mannitol mixture for 48 h. The control group consisted of cultures treated with NG. Cell viability was assessed using the MTT assay. Data were presented in histograms and expressed as mean ± SEM (*n* = 5, in triplicate). * *p* < 0.05 vs. NG group at 24 h; ^a^ *p* < 0.05 vs. NG group at 48 h; ^b^ *p* < 0.05 vs. NG group at 72 h. (**c**–**e**) NG-cultured cells were untreated or treated with a series of HG (25–125 mM) for 24 h. The levels of pp53, p53, Bcl-2, cleaved caspase-3, LC3B, and p62 were analyzed by Western blotting. The blot collection in (**d**) was obtained from the same membrane that underwent stripping/reprobing. Quantitative results were presented in histograms. Densitometric analysis values are expressed as mean ± SEM (*n* = 4). * *p* < 0.05 vs. NG group’s Bcl-2; ^a^ *p* < 0.05 vs. NG group’s cleaved caspase-3; ^b^ *p* < 0.05 vs. NG group’s LC3B-II; ^c^ *p* < 0.05 vs. NG group’s p62.

Hyperglycemia is a hallmark of diabetes, and glucotoxicity is a major cause of diabetic complications including dementia [24]. Acute hyperglycemia adversely affects innate immunity [25]. We therefore examined if HG induces gliotoxicity in BV-2 microglia to weaken neuroimmunity. HG reaching 100 mM started to injure BV-2 cells within the initial 24 h compared to the control group exposed to NG (Figure 2a). A series of mannitol mixtures consisting of 5.5 mM glucose and different mannitol concentrations (19.5–119.5 mM) served as an osmotic control and produced no cytotoxic effect within 48 h (Figure 2b), suggesting that hyperosmolarity is not the cause to compromise cell viability.

Tumor suppressor p53 is involved in cell death, including apoptosis and autophagy [26,27], so we assessed the expression of active p53 and markers representing apoptosis and autophagic degradation. Glucose induced a concentration-dependent increase in phosphorylated p53 levels, indicating that HG activates p53 signaling (Figure 2c). HG also progressively induced apoptosis and autophagic degradation activity, as evidenced by decreased B-cell lymphoma 2 (Bcl-2) levels, increased caspase-3 cleavage, elevated light chain 3B (LC3B)-II levels, and reduced p62 expression (Figure 2d,e). Thus, HG triggers p53-associated glial cytotoxicity and subjects BV-2 microglia to apoptosis and autophagic degradation. Glucotoxicity resulting from hyperglycemia in the brain may threaten the survival of microglial cells and compromise their innate immune defense functions.

### 3.3. Cana Rescues BV-2 Microglia from HG-Induced Apoptosis and Autophagic Degradation

**Figure 3:**
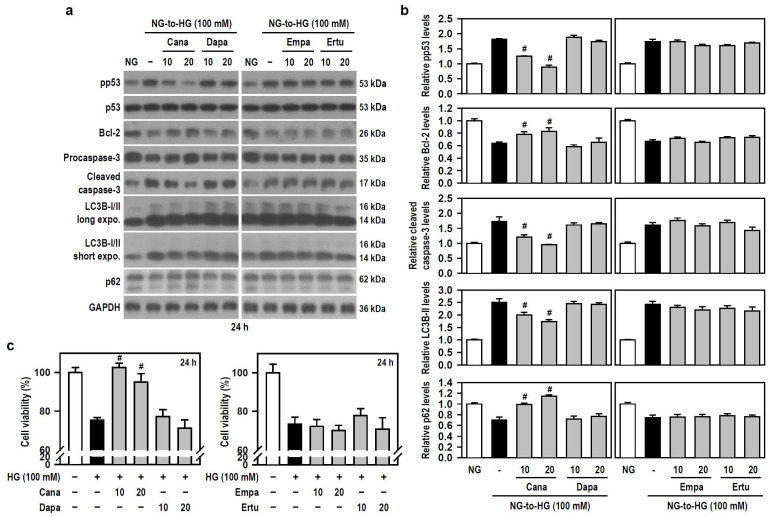
Cana protects BV-2 cells from HG-induced gliotoxicity. (**a**,**b**) NG-cultured cells, with or without the four SGLT2 inhibitors (10 and 20 μM) given 30 min in advance, were stimulated with HG (100 mM) for 24 h. Cultures treated with NG served as the control group. Western blotting was used to analyze the levels of pp53, p53, procaspase-3, cleaved caspase-3, LC3B, and p62. The blot collection was obtained from the same membrane that underwent stripping/reprobing. Densitometric analysis values for quantified proteins (i.e., pp53, Bcl-2, cleaved caspase-3, LC3B-II, and p62) were shown using histograms and are expressed as mean ± SEM (*n* = 4). ^#^ *p* < 0.05 vs. HG group. (**c**) NG-cultured cells were exposed to 100 mM HG for 24 h, either in the presence or absence of each indicated SGLT2 inhibitor. The results of cell viability were presented using histograms. Percent values are expressed as mean ± SEM (*n* = 5, in triplicate). ^#^ *p* < 0.05 vs. HG group.

Along with acting as antidiabetic agents by inhibiting glucose uptake in the kidney, SGLT2 inhibitors exhibit beneficial cognitive effects in diabetic patients and animal models of dementia [17,18,19,20]. Thus, we examined if SGLT2 inhibitors alleviates HG-induced BV-2 cell damage. Based on the results of Figure 2a,e, we used 100 mM HG to attack cells. Compared with HG alone, Cana, but not the other three SGLT2 inhibitors, significantly counteracted apoptosis- and autophagy-related signaling cascades activated by HG (Figure 3a,b). This effect was evidenced by reduced phosphorylation of p53, decreased levels of cleaved caspase-3 and LC3B-II, and increased expression of Bcl-2 and p62. Further, compared to cultures exposed to HG alone, a significant retention of cell viability under HG condition was found only in Cana-treated cultures (Figure 3c), indicating that Cana inhibits apoptotic death and autophagic degradation, thereby protecting BV-2 microglia against HG-induced gliotoxicity.

### 3.4. Cytoprotective Effect of Cana against HG Toxicity Is Not Mediated through Induction of Heme Oxygenase 1 (HO-1) and/or Heat Shock Protein 70 (HSP70)

Glucose fluctuations, such as acute hyperglycemia, are recognized as a stress that can significantly alter microglial activity [11]. Stress responses are typically mediated by stress-inducible proteins. Both HO-1 (HSP32) and HSP70 were chosen for their cytoprotective effects in many experimental models, including hyperglycemic cerebral ischemia and oxidative stress-induced gliotoxicity [28,29]. Thus, we investigated if SGLT2 inhibitors modulate the induction of HO-1 and HSP70 in BV-2 microglia exposed to HG. Compared to the NG group, HG caused a significant upregulation of HO-1 and HSP70 levels, although HO-1 levels decreased slightly with increasing HG concentrations (Figure S2a,b). Moreover, compared to the group of HG alone, no significant changes in both HO-1 and HSP70 levels were found in each group of SGLT2 inhibitor plus HG (Figure S2c,d), suggesting that none of the tested SGLT2 inhibitors modulate the induction of these two HSPs by HG, and that the cytoprotective effect of Cana against HG-induced gliotoxicity is not mediated through HO-1 and/or HSP70.

### 3.5. Cana Attenuates HG-Induced Oxidation and Inflammation in BV-2 Microglia

**Figure 4:**
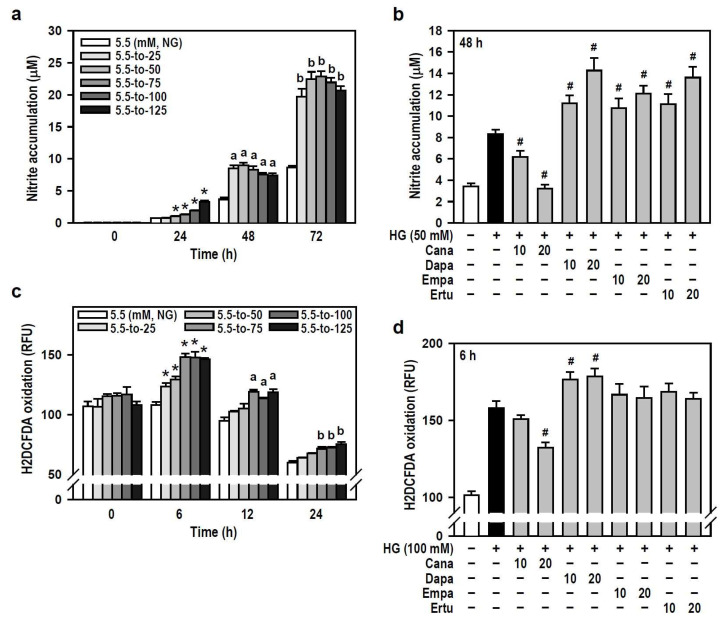
Cana reduces the production of oxygen free radicals induced by HG in BV-2 cells. (**a**,**b**) NG-cultured cells were exposed to a range of HG treatments (25–125 mM) for varying and independent durations, as indicated. Alternatively, cells were stimulated with HG (50 mM) for 48 h, with or without the administration of the four SGLT2 inhibitors (10 and 20 µM) given 30 min in advance. Nitrite accumulation was measured using a Griess reaction-based assay to evaluate NO production. Data were shown using histograms and are expressed as mean ± SEM (*n* = 5, in triplicate). * *p* < 0.05 vs. NG group at 24 h; ^a^ *p* < 0.05 vs. NG group at 48 h; ^b^ *p* < 0.05 vs. NG group at 72 h. ^#^ *p* < 0.05 vs. HG group. (**c**,**d**) The same treatments as (**a**,**b**) were administered, and the cultures were incubated for varying and independent durations as indicated (**c**), or only for 6 h (**d**). The fluorogenic probe H_2_DCFDA was used to monitor intracellular ROS production. Results are displayed as histograms and data are expressed as mean ± SEM (*n* = 5, in triplicate). RFU: relative fluorescence unit. * *p* < 0.05 vs. NG group at 6 h; ^a^ *p* < 0.05 vs. NG group at 12 h; ^b^ *p* < 0.05 vs. NG group at 24 h. ^#^ *p* < 0.05 vs. HG group.

Hyperglycemia has been reported to induce oxidative stress and neuroinflammation, leading to cognitive impairment in diabetics [2,30]. We therefore examined if SGLT2 inhibitors are effective against HG-induced oxidative and inflammatory responses in BV-2 microglia. In terms of oxygen free radical levels, HG did induce concentration- and time-dependent NO production compared to the NG group, even though a slight decline in productive NO levels was observed at 48 or 72 h under higher HG (≥100 mM) conditions (Figure 4a). Of the four SGLT2 inhibitors, Cana effectively blocked HG-induced NO production while the others enhanced the HG effect instead (Figure 4b). Also, HG (25–125 mM at 6 h) caused a concentration-dependent increase in intracellular ROS levels which later gradually decayed over time (Figure 4c). Only Cana markedly repressed the effect of HG on ROS production compared to HG alone (Figure 4d). Thus, Cana is better than the other three SGLT2 inhibitors to relieve ROS/NO-driven oxidative stress.

**Figure 5:**
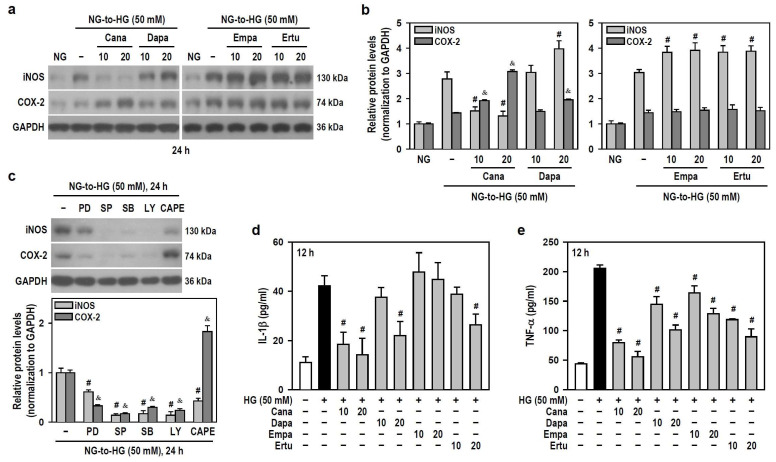
Cana suppresses the induction of iNOS and the secretion of inflammatory cytokines IL-1β and TNF-α in HG-treated BV-2 cells but augments the expression of COX-2. (**a**,**b**) NG-cultured cells, with or without the four SGLT2 inhibitors (10 and 20 μM) given 30 min in advance, were stimulated with HG (50 mM) for 24 h. Cultures treated with HG served as the control group. The iNOS and COX-2 levels were analyzed using Western blotting. The relatively densitometric analysis results were shown using histograms, and values are expressed as mean ± SEM (*n* = 4). ^#^ *p* < 0.05 vs. HG group’s iNOS; ^&^ *p* < 0.05 vs. HG group’s COX-2. (**c**) NG-cultured cells, with or without specific signaling pathway inhibitors (20 μM PD98059 (PD), 10 μM SP600125 (SP), 15 μM SB203580 (SB), 10 μM LY294002 (LY), and 3 μM CAPE) given 30 min in advance were exposed to 50 mM HG for 24 h. After Western blotting detection, the relatively densitometric analysis results of iNOS and COX-2 levels were shown using histograms, and values are expressed as mean ± SEM (*n* = 4). ^#^ *p* < 0.05 vs. HG group’s iNOS; ^&^ *p* < 0.05 vs. HG group’s COX-2. (**d**,**e**) The same treatments as (**a**,**b**) were administered, and the cultures were incubated only for 12 h. The secretion levels of IL-1β and TNF-α were assayed using ELISA. Results were shown using histograms and values are expressed as mean ± SEM (*n* = 4, in triplicate). ^#^ *p* < 0.05 vs. HG group.

As for the induction and secretion of proinflammatory factors, HG (≥25 mM) upregulated the protein levels of inducible nitric oxide synthase (iNOS) and cyclooxygenase-2 (COX-2) (Figure S3). Only Cana significantly reduced the effect of HG on iNOS induction, while the others enhanced HG-induced iNOS production instead. Also, both Cana and Dapa did augment the HG effect on COX-2 induction, while neither Empa nor Ertu showed such induction (Figure 5a,b). To explore if SLGT2 inhibitors influence HG-induced inflammatory factors by targeting specific pathways, we employed signaling pathway inhibitors. PD98059, SP600125, SB203580, and LY294002 suppressed HG-induced iNOS and COX-2 production to differing extents, however, only CAPE lessened HG-induced iNOS but increased the HG effect on COX-2 induction (Figure 5c). These clues indicate that, similar to the action of CAPE, both Cana and Dapa enhance the effect of HG on COX-2 induction by inhibiting NFκB activity. Additionally, Empa only reduced TNF-α secretion induced by HG, while the others effectively inhibited HG-stimulated secretion of both IL-1β and TNF-α to varying degrees (Figure 5d,e). Not Empa, but the other three significantly reduced HG-induced expression of the NLR family pyrin domain containing 3 (NLRP3), an inflammasome sensor (Figure S4a,b).

Thus, in HG-stimulated BV-2 microglia, Cana negatively modulates the levels of all tested proinflammatory factors and functions as an NFκB inhibitor. Among the four SGLT2 inhibitors, Cana exhibits superior anti-inflammatory efficacy under HG conditions.

### 3.6. Cana Inhibits HG-Stimulated NFκB, JNK, p38 and Akt Activities in BV-2 Microglia

We next explored signal cascades that might correspond to the aforementioned results. NFκB is a main transcription factor for microglial immune function [31,32]. MAPKs, another major inflammatory signal, participate in stress-related cell immunity and self-degradation, while PI3K/Akt is related to cell survival and serves as a predominant pathway for the synthesis and production of proinflammatory mediators [32,33,34]. Thereupon, whether SGLT2 inhibitors affect NFκB activity and the phosphorylation state of ERK, JNK, p38, and Akt was examined in BV-2 cells exposed to acute shifts from NG to HG.

**Figure 6:**
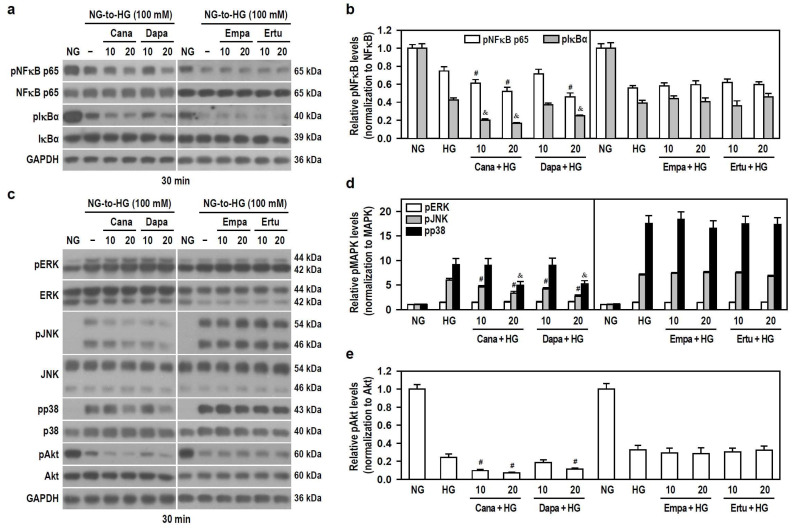
Both Cana and Dapa decrease the phosphorylation levels of JNK, p38, Akt, and NFκB in BV-2 cells treated with HG. NG-cultured cells, with or without the four SGLT2 inhibitors (10 and 20 μM) given 30 min in advance, were treated with 100 mM HG for 30 min. NG-treated cultures served as a control group. Protein levels were analyzed using Western blotting. (**a**,**b**) The levels of pNFκB p65, NFκB p65, pIκBα and IκBα were detected. Alternatively, in (**c**–**e**) the levels of pERK, ERK, pJNK, JNK, pp38, p38, pAkt and Akt were determined. The blot collection in each individual panel was obtained from the same membrane that underwent stripping/reprobing. The relatively densitometric analysis results were shown using histograms and are expressed as mean ± SEM (*n* = 4). In (**b**), ^#^ *p* < 0.05 vs. HG group’s pNFκB p65 and ^&^ *p* < 0.05 vs. HG group’s pIκBα; in (**d**), ^#^ *p* < 0.05 vs. HG group’s pJNK and ^&^ *p* < 0.05 vs. HG group’s pp38; in (**e**), ^#^ *p* < 0.05 vs. HG group.

HG concentration-dependently decreased the phosphorylation levels of NFκB p65 and IκBα (Figure S5a,b), indicating that acute HG exposure reduces NFκB activity. To ensure a significant HG stimulation, 100 mM was used. Only Cana and Dapa effectively increased the downregulated effect of HG on the phosphorylation of NFκB p65 and IκBα (Figure 6a,b), implying that both Cana and Dapa could reduce NFκB activity under acute HG conditions. Also, the translocation of NFκB p65 was characterized using IF staining and nuclear/cytoplasmic fractionation. Qualitatively, NFκB p65 was evenly distributed in both cytoplasm and nucleus of untreated cells. Interestingly, NFκB p65 was mostly detectable in the cytoplasm of HG-treated cells (Figure S6a), suggesting that acute HG promotes the translocation of NFκB p65 to cytoplasm. In comparison to HG-stimulated cells, the appearance of NFκB p65 within the nucleus was further reduced, but it was increased in the cytoplasm after combined treatments of HG with Cana or Dapa (Figure S6b), implying that both Cana and Dapa potentiate the translocation of NFκB p65 to the cytoplasm, thereby attenuating NFκB activity under acute HG conditions.

In addition, HG significantly increased the phosphorylation levels of ERK, JNK and p38, but decreased Akt phosphorylation in a concentration-dependent manner (Figure S5c–e), meaning that acute HG activates MAPKs but lessens Akt signaling. To ensure that HG drives significant ERK activation, 100 mM HG was used to activate all kinases. All SGLT2 inhibitors had no effect on HG-induced ERK phosphorylation. Cana and Dapa, but not Empa and Ertu, effectively reduced HG-induced JNK and p38 phosphorylation and increased the downregulated effect of HG on Akt phosphorylation (Figure 6c–e), suggesting that both Cana and Dapa lessen JNK, p38, and Akt activities stimulated by acute HG.

Therefore, at the same concentration levels, the finding that neither Empa nor Ertu affects these signaling pathways highlights the extra pleiotropy and specific targeting of Cana and Dapa on microglia. Cana may be better than Dapa to inhibit the activity of NFκB, JNK, p38 and PI3K/Akt pathways under conditions of acute hyperglycemia.

### 3.7. Cana-Elicited Inhibition of HG Toxicity Is Not Mediated by Modulating SGLT2 Expression

**Figure 7:**
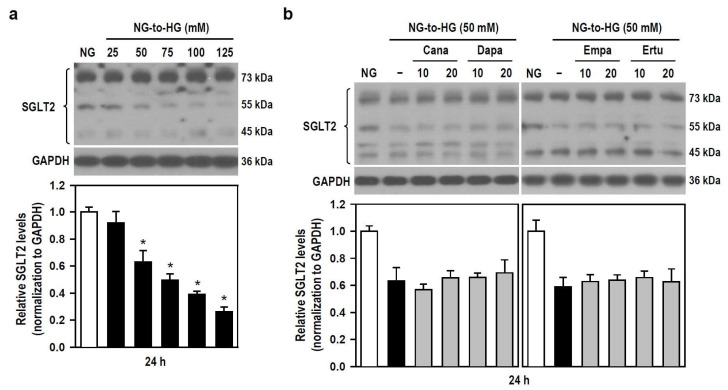
SGLT2 inhibitors show no effect on HG-induced decreases in SGLT2 protein levels in BV-2 cells. (**a**) NG-cultured cells were untreated or treated with a series of HG (25–125 mM) for 24 h. (**b**) Alternatively, cells were stimulated with 50 mM HG for 24 h, with or without the four SGLT2 inhibitors (10 and 20 μM) given 30 min in advance. NG-treated cultures served as the control group. Western blotting was used to analyze SGLT2 protein levels. Quantitative results were displayed as histograms. Densitometric analysis values are expressed as mean ± SEM (*n* = 4). * *p* < 0.05 vs. NG group.

The pharmacological mechanism by which SGLT2 inhibitors alleviate HG toxicity (i.e., cytotoxicity and neuroinflammation) is unclear, so we examined if SGLT2 inhibitors affect the expression level of SGLT2 in BV-2 cells treated with HG. With the gradual increase of HG concentration, the expression level of SGLT2 gradually decreased, reaching significance when HG ≥ 50 mM (Figure 7a). None of all SGLT2 inhibitors altered the HG-triggered reduction in SGLT2 expression levels (Figure 7b). The results indicate that HG-induced inflammatory toxicity is associated with reduced SGLT2 protein level in BV-2 microglia. Moreover, Cana fails to counteract this, meaning that SGLT2 does not mediate the inhibitory effect of Cana on HG insults.

## 4. Discussion

A growing body of preclinical and now clinical studies have found neuroprotective effects of drugs approved for diabetes [17,35,36], however, their anti-neurodegenerative efficacy in clinical settings still needs to be elucidated. Brain inflammation characterizes many chronic neurodegenerative diseases, including diabetic dementia and other forms of cognitive decline. The pathological mechanisms of hyperglycemia-related brain diseases are highly complicated and involve cell death/degradation and production/secretion of inflammatory factors during the neuroinflammatory process. This is the first study demonstrating that Cana is more effective to alleviate HG-induced inflammatory toxicity in BV-2 microglia through a side-by-side in vitro comparison of four SGLT2 inhibitors. This model perhaps explains how Cana reduces hyperglycemia-related neuropathy.

The neuroprotective effects of SGLT2 inhibitors are supported by preclinical studies related to cognitive decline and dementia [17,37,38]. Nevertheless, the precise mechanisms by which SGLT2 inhibitors achieve neuroprotection remain poorly understood. A comorbid association between neurodegeneration and T2DM implies that there may be shared pathophysiological mechanisms. Several putative “shared pathways” have been revealed through comparative analyses, including signaling pathways for neurotrophins, PI3K/AKT, AMPK/mTOR, and MAPKs, and pathway crosstalk [39,40,41]. Accordingly, some proof-of-concept studies using SGLT2 inhibitors have attempted to cure diabetes-related brain degeneration. Empa prevented cognitive decline in obese and T2DM mice, which was associated with cerebral oxidative stress attenuation and increases in cerebral brain-derived neurotrophic factor [20]. Dapa improved hippocampal synaptic plasticity and prevented cognitive decline by restoring the phosphorylation levels of the insulin receptor, Akt/PKB, and NFκB p65 in HFD-induced obese rats [22]. Herein we propose that Cana has the best anti-inflammatory efficacy among four SGLT2 inhibitors at similar concentrations by modulating the effects of HG on NFκB, JNK, p38 and Akt signaling pathways. Particularly, Cana mimics CAPE as an NFκB inhibitor. Sequentially, Cana mitigates HG-induced apoptosis (i.e., cell self-destruction due to environmental maladaptation), autophagy (i.e., degradation of unnecessary or dysfunctional cellular structures to adapt to the environment), oxidative stress, and inflammation in BV-2 microglia.

The anti-inflammatory effects of SGLT2 inhibitors are widely proposed. Dapa suppressed collagen synthesis by increasing activation of M2 macrophages (an anti-inflammatory subgroup) and inhibiting myofibroblast differentiation during the acute phase after myocardial infarction in rats [42]. In diabetic rat models, Empa reduced renal expression of proinflammatory cytokines and chemokines, including TNF-α, urinary markers of renal inflammation such as IL-6, and apoptosis [43]. In a model of diabetes-related neurodegenerative disease, Dapa exerted anti-apoptotic and anti-inflammatory effects by regaining normal levels of bax, bcl-2, and phosphorylated NFκB p65 expression in HFD-induced obese rats [22]. Empa prevented impaired cognitive function in *db*/*db* mice, which was associated with attenuation of cerebral oxidative stress [20]. The Cana effect on the suppression of obesity-related inflammation in the nervous system of HFD-fed obese mice was mediated by reducing the levels of proinflammatory biomarkers and cytokines (e.g., Iba1 and IL-6) and the accumulation of macrophages [21]. Nevertheless, these in vivo animal studies failed to account for the independent and direct anti-inflammatory effects of SGLT2 inhibitors in distinct or individual brain cell populations. We applied an in vitro cell model to simulate hyperglycemia and found that Cana achieves a direct anti-inflammatory effect in BV-2 microglia exposed to HG by increasing the expression of Bcl-2 and p62 and decreasing the levels of LC3B-II, cleaved caspase-3, activated p53 and proinflammatory factors (i.e., NO, ROS, iNOS, NLRP3, IL-1β and TNF-α). These efficacies depend on neither HSPs (such as HO-1 and HSP70) nor SGLT2.

In this study, NFκB p65 translocation is of concern. We found that HG alone or HG plus an SGLT2 inhibitor (Cana or Dapa) induced NFκB p65 translocation to cytoplasm, showing that HG reduces NFκB activity. This could be proved by another finding (Figure 5d), i.e., a NFκB activation inhibitor CAPE promoted rather than inhibited the effect of HG on COX-2 induction, indicating that HG induces COX-2 expression by suppressing NFκB activation. Further, it is well-reported that NFκB activation by immune activator lipopolysaccharide (LPS) is directly regulated via phosphorylation of PI3K/Akt, which is the main upstream molecule of NFκB [44]. The PI3K/Akt/NFκB signaling axis has been proposed by several other studies using BV-2 microglia [45,46,47,48]. In agreement with these studies, we did find that the reduction of Akt phosphorylation in response to HG stimulation showed the same trend as that of NFκB p65. We also confirmed that NFκB activity was clearly inhibited by an Akt inhibitor MK2206, consistent with the results obtained with HG stimulation alone (Figure S7). These clues indicate that HG reduces PI3K/Akt-dependent NFκB activation, impairing NFκB translocation to the nucleus. Of note, the existence of PI3K/Akt/NFκB axis in this cell model shows no conflict with the findings that Cana and Dapa reduce NFκB activity under HG conditions, since we also noted the inhibition of HG-stimulated Akt activity by Cana and Dapa and the consequent decrease in NFκB activity.

Recently, a homogeneous study proposed by Heimke et al. showed that Empa exerts anti-inflammation by interacting with Na^+^/H^+^ exchanger 1 (NHE-1), but not SGLT2, as well as by inhibiting the ERK1/2 and NFκB pathways in LPS-activated primary microglia [23]. Compared with their study, ours is unique in that cells were challenged with HG instead of LPS in order to evaluate the anti-inflammatory effects of distinct antihyperglycemic drugs rather than a single one. Regarding Empa concentration used in experiments, the 50 µM they chose was higher than ours, perhaps implying that neither 10 nor 20 μM is the optimal working concentration of Empa in our cell model. Consistent with their view, the anti-inflammatory effect of SGLT2 inhibitor is associated with inhibition of NFκB. This is mediated not through interactions with SGLT2 but rather through an off-target effect. In short, the strength of the present study is to provide a comprehensive comparison between four inhibitors, albeit using only a microglial cell line.

We found that Cana and Dapa could block the proliferation of BV-2 microglia, which may be one of the means to alleviate inflammation. While we do not know if this effect is concentration-dependent, Cana’s antiproliferative concentrations could reach 10 μM, which is well within clinically achievable levels in the plasma of patients taking this SGLT2 inhibitor [49]. This proliferation inhibition by Cana was not secondary to cell injury or death, suggesting that Cana acts cytostatically rather than cytotoxically. This finding agrees with previous in vitro studies pointing out Cana blockades of the proliferation of vascular smooth muscle cells, renal proximal tubule epithelial cells, endothelial cells, and various tumor cells [50,51,52,53], suggesting that Cana inhibits proliferation regardless of cell type. Also, its antigrowth activity has been verified in vivo in murine cancer models [53,54]. Cana’s notable ability speaks to its superiority in terms of affinity or structure specificity, since pharmacologically parallel comparisons showed the other three failing to inhibit BV-2 cell proliferation at a concentration of 10 μM.

The excellent anti-inflammatory efficacy of Cana over the other three SGLT2 inhibitors in our model may be due to its specificity in molecular structure that exhibits the highest affinity for the same target. For example, apart from acting selectively on SGTL2, these inhibitors also affect SGLT1 to various extents. The half-maximal inhibitory concentration (IC50) of Cana for SGLT1 is 710 nM, while Dapa, Empa, and Ertu have an IC50 for SGLT1 of 1400, 8300, and 1960 nM, respectively [55]. In other words, among the four SGLT2 inhibitors, Cana has the greatest potential for inhibiting SGLT1, whereas Empa is the most selective for SGLT2 and has the lowest potential for interaction with SGLT1. In this regard, we speculate that SGLT1 inhibition must be associated with anti-inflammatory efficacy in this study, since the fact that the IC50 of Cana is theoretically superior to that of Dapa, Empa, and Ertu perfectly matches what we observed in Cana-driven efficacy against HG-induced inflammation.

Another suspect targeted non-specifically by SGLT2 inhibitors may be NHE-1. NHE-1 mediating H^+^ extrusion during “respiratory bursting” has been considered important for microglial activation [56]. NHE-1 inhibition reduced microglial activation, NADPH oxidase activity, and proinflammatory responses in a mouse model of transient focal cerebral ischemia/reperfusion [57]. It has been reported that cardiac NHE-1 inhibition by SGLT2 inhibitors is not a drug-specific effect but a common class effect, showing similar inhibitory potential and binding affinity for NHE-1 by Cana, Empa, and Dapa [58]. Regarding this, NHE-1 might be activated under HG conditions and Cana might inhibit NHE-1. However, as we found different inhibitory profiles of Cana, Dapa, Empa, and Ertu on HG-induced inflammatory toxicity, NHE-1 inhibition could not explain Cana being the drug with broad anti-inflammatory effects in the present model.

Among HSPs, HO-1 and HSP70 have received particular attention for their neuro-protective effects against apoptosis, oxidative stress, and inflammation in various brain disease models [59,60]. Thus, induction of HSPs such as HO-1 and HSP70 by some vanguard drugs may help to explain their pharmacological mechanisms for treating neurological diseases [61,62]. In this study, both HO-1 and HSP70 were well upregulated upon HG stimulation, however, such induction was not modulated by any of the SGLT2 inhibitors tested, implying that Cana’s cytoprotective effect is dependent on neither HO-1 nor HSP70. This result was inconsistent with studies which present that Cana stimulates HO-1 expression, thereby inhibiting vascular smooth muscle cell (SMC) proliferation and migration and promoting antioxidative and anti-inflammatory effects in cardiomyocytes [50,63]. There are several conjectures as to the reasons for this discrepancy: 1) Cana-induced HO-1 is masked by robust HG induction, resulting in no detectable synergism; 2) regardless of whether Cana is added or not, the maximally induced level of HO-1 is completed by HG; 3) the characteristics of distinct cell lines are involved. Notably, Cana-triggered ROS was responsible for inducing HO-1 expression in vascular SMCs [50], however, this phenomenon was not observed in our cultured BV-2 cells. Also, the discrepancy may be due to different in vitro culture systems. Little is known about the relationship between HSP70 and SGLT2 inhibitors. A study showed the failure of Dapa to affect HSP70 levels in LPS-stimulated mouse cardiac fibroblasts [64], which is similar to our findings.

We observed that SGLT2 protein levels gradually decreased with increasing glucose concentrations, although SGLT2 upregulation was reported in HG-treated mouse mesangial cells, kidney tissue of *db*/*db* or HFD-induced obese mice, and renal proximal tubular cells collected from T2DM patients [65,66,67,68]. Studies using human kidney biopsies have yielded inconsistent results. Some reported unchanged SGLT2 expression in kidney tissue taken from diabetic patients compared with nondiabetic controls, while others reported reduced SGLT2 expression [69,70]. As far as we know, no other study has examined SGLT2 protein expression in central immune cells exposed to glucose fluctuations. Even though diabetes-induced changes in SGLT2 expression and/or activity remain to be verified, inhibition of SGLT2 to confer antioxidative and anti-inflammatory effects is well documented. Thus, in our view, adaptive downregulation of SGLT2 protein reduces more glucose uptake as compensatory feedback to suppress BV-2 cell proliferation and HG-induced inflammatory stress.

In agreement with previous studies [66,71], we detected no differential SGLT2 ex-pression between the groups of HG alone and HG plus SGLT2 inhibitors, and also between the NG group and each SGLT2 inhibitor group (Figure S8), suggesting the incapacity of Cana to modulate the effects of HG on SGLT2 protein and Cana’s cytoprotection against HG toxicity being not mediated by SGLT2. It may be that Cana modulates HG-triggered alterations in NFκB, PI3K/Akt, JNK and p38 activities by inhibiting SGLT1/2 activity rather than altering SGLT2 gene/protein expression or interacts directly with other mediators to link intracellular signal cascades. Evidence has shown that the actions of SGLT2 inhibitors do involve NFκB, JNK, p38, and PI3K/Akt signaling, however, the details of how these signaling cascades are regulated remain unclear. Most studies simply propose associations without providing more detailed evidence on molecular interactions. Abdollahi et al. proposed that regardless of glucose concentrations, Dapa exerted direct anti-inflammatory effects, at least partly, by inhibiting Toll-like receptor 4 (TLR4) expression and activation of NFκB along with the secretion of pro-inflammatory mediators [72]. Empa improved the expression of phosphorylated Akt and p38 in cardiac stromal cells under HG conditions [73]. Similar to ours on modulating signaling pathways, the renoprotective effect of Cana depended on reduced uptake of cisplatin in kidney and Akt activation. This protection was also achieved by Cana reducing cisplatin-induced phosphorylation of p53, p38, and JNK, but not ERK1/2 [74]. Since SGLT2 serves not as intracellular signal transmitters like enzymes, no clear evidence shows that SGLT2 directly regulates NFκB, JNK, p38, and PI3K/Akt pathways. Nevertheless, SGLT2 may affect the activity of various signal cascades through relevant mediators. Upregulation of SGLT2 caused glomerular injury mainly via the IGF1R/PI3K pathway [75]. Ashrafi et al. reported that HG-related SGLT2 activation promoted nuclear–cytoplasmic translocation of high mobility group box 1 (HMGB1), which in turn activated the TLR2/4 pathway in renal tubules and glomeruli, whereas SGLT2 inhibitors reduced TLR2/4 expression [43,76]. This implies a link between SGLT2 and TLR2/4. HMGB1 released from necrotic cells activated ERK1/2, JNK and p38 cascade reactions by binding to TLR2/4 and RAGE, further promoting the expression of NFκB to aggravate inflammatory response [77]. These relevant molecular linkages suggest the existence of SGLT2 signaling to multiple immune/inflammatory pathways. In addition, inhibition of SGLT2 by Cana reportedly reduced SGLT2 protein expression in kidney tissues of *db*/*db* or HFD-fed obese mice [67,68]. Thus, comprehensive analyses of the interaction between Cana and SGLT2 protein in brain tissues/cells of diabetic models will be future research directions.

An acute shift from NG to HG surely accompanies the change of osmolality. Therefore, high mannitol was included as a control of hyperosmolarity (5.5 mM glucose plus 94.5 mM mannitol) to test the impact of osmotic fluctuations per se on the induction of inflammation and the anti-inflammatory efficacy of Cana. In this study, high mannitol-induced hyperosmolarity failed to copy significant NFκB activity changes we observed in BV-2 cultures that underwent an acute NG–HG shift (Figures S5a and S9a). Despite inducing inflammation, hyperosmolarity was unable to elicit significant cell lethality. Notably, Cana still exhibited anti-inflammatory efficacy in BV-2 cells by modulating the activities of NFκB, MAPKs, and Akt, which is another inhibitory mode different from counteracting HG-induced inflammation (Figure 3, Figure 5, Figure 6 and Figure S9). Thus, Cana may be effective in addressing inflammation caused by hyperglycemia and hyperosmotic stress. In addition, to enhance the persuasiveness and credibility of this study, several key experiments testing the anti-inflammatory efficacy of Cana were conducted again under lower HG (25 mM) conditions, and the results showed that Cana’s efficacy in inhibiting inflammation remains reproducible and significant, even despite the lack of substantial cell death/damage caused by apoptosis and autophagic degradation (Figures S10).

About the weakness of this in vitro study, we only examined the direct effects of four SGLT2 inhibitors at the same concentration levels against HG-induced inflammatory gliotoxicity. We cannot rule out the possibility that Dapa, Empa, and Ertu attenuate HG insults to varying degrees at other concentrations above or below the current settings (10 and 20 μM). For example, Cana at 10 μM, while Empa and Dapa at 1 and 0.5 μM, respectively, are considered clinically relevant concentrations. The concentration-response effect of each SGLT2 inhibitor should be evaluated to select the optimal concentrations for exploring anti-inflammatory effects in future microglia models.

Regarding the limitations of this study, despite confirming that HSPs (HO-1 and HSP70) and SGLT2 are not involved in mediating the anti-inflammatory effects of SGLT2 inhibitors, we were unable to identify potential targets that might directly interact with SGLT2 inhibitors. Additionally, the use of 100 mM HG is non-physiological, potentially limiting the generalizability and translational impact of our results. Future studies should consider employing HG concentrations closer to real physiological conditions, such as 20–30 mM to improve the reliability and application value of the findings. Another limitation is that only mouse BV-2 microglia were used, even though this type of brain immune cell line is the most commonly used to explore CNS immunity. Hence, the transferability of results might be problematic. The clinical practicality is limited, making it difficult to match the situation of human studies. Considering this, more comprehensive studies using primary microglial cultures and in vivo animal T2DM models should be a future priority to validate the present findings, as well as translate and understand drug effects in patients using SGLT2 inhibitors.

## 5. Conclusions

We identified Cana as the best of four SGLT2 inhibitors at the same concentration levels against HG-induced inflammatory toxicity in BV-2 microglia (Figure S11). The mechanism by which Cana attenuates HG-induced apoptosis, autophagic degradation, oxidation, and inflammation is mainly through modulating HG-stimulated NFκB, JNK, p38 and PI3K/Akt pathway activities. However, HSPs (i.e., HO-1 and HSP70) and SGLT2 fail to mediate Cana-driven protection. Cana also reduces microglial proliferation. It was therefore concluded that Cana is capable of counteracting hyperglycemia-related inflammatory toxicity to improve microglial viability. Past epidemiological and clinical reports have noted that SGLT2 inhibitors alleviate cognitive decline in patients with diabetes and AD. Our findings support the clinical efficacy of SGLT2 inhibitors, especially Cana, in anti-neurodegeneration.

## Data Availability

The data that support the findings of this study are available within the article.

## Supplementary Materials

The following supporting information can be downloaded at https://www.mdpi.com/article/10.3390/biomedicines12010036/s1. Supplementary Materials and Methods include antibodies, BrdU labeling for in vitro cell proliferation assay, and Western blotting and subcellular fractionation; Figure S1: Two additional replicates of the in vitro cell proliferation assay conducted using BrdU labeling; Figure S2: All tested SGLT2 inhibitors show no impact on the HG-induced expression of HO-1 and HSP70 in BV-2 cells; Figure S3: HG induces iNOS and COX-2 expression in BV-2 cells in a concentration-dependent manner; Figure S4: HG stimulation leads to an upregulation of NLRP3 expression, which is effectively inhibited by Cana, Dapa, and Ertu, but not Empa; Figure S5: HG concentration-dependently alters the activity of NFκB, MAPKs, and Akt in BV-2 cells; Figure S6: Both Cana and Dapa further increase the translocation of HG-induced NFκB p65 to the cytoplasm of BV-2 cells; Figure S7: The phosphorylated NFκB p65 level in BV-2 cells is reduced by the Akt inhibitor MK2206; Figure S8: SGLT2 inhibitors fail to induce any changes in the levels of SGLT2 protein in NG-cultured BV-2 cells; Figure S9: Although high mannitol triggers immune inflammation not by altering NFκB activity and fails to elicit significant cellular lethality, Cana still shows anti-inflammatory efficacy in BV-2 cells by modulating the activities of NFκB, MAPKs, and Akt; Figure S10: Cana alleviates immune inflammation and associated signaling cascades induced by 25 mM HG in BV-2 cells, even despite the lack of substantial cell death/damage caused by apoptosis and autophagic degradation; Figure S11: A summary illustration is provided to demonstrate how four SGLT2 inhibitors protect BV-2 microglia from HG-induced inflammatory toxicity.
