# Supplementary material for: SGLT2 Inhibitor Canagliflozin Alleviates High Glucose-Induced Inflammatory Toxicity in BV-2 Microglia
Source: Biomedicines. 2023 Dec 22;12(1):36. doi: 10.3390/biomedicines12010036 (PMC10813070; doi:10.3390/biomedicines12010036)
Supplement: Supplementary file 1 [file biomedicines-12-00036-s001.zip › biomedicines-2705894-supplementary.pdf]

Supplementary Materials

Title

SGLT2 Inhibitor Canagliflozin Alleviates High Glucose-Induced Inflammatory Toxicity in BV-2 Microglia

Author list and affiliations

Ching-Tien Lee [1], Kun-Der Lin [2], Cheng-Fang Hsieh [3] and Jiz-Yuh Wang [4,5,6,7,*]

[1] Department of Medical and Healthcare Business, Hsin-Sheng College of Medical Care and Management, Taoyuan 32544, Taiwan

[2] The Lin's Clinic, Kaohsiung 80778, Taiwan

[3] Division of Geriatrics and Gerontology, Department of Internal Medicine, Kaohsiung Medical University Hospital, Kaohsiung 80756, Taiwan

[4] Graduate Institute of Medicine, College of Medicine, Kaohsiung Medical University, Kaohsiung 80708, Taiwan

[5] Neuroscience Research Center, Kaohsiung Medical University, Kaohsiung 80708, Taiwan

[6] Research Center for Precision Environmental Medicine, Kaohsiung Medical University, Kaohsiung 80708, Taiwan

[7] Department of Medical Research, Kaohsiung Medical University Hospital, Kaohsiung 80756, Taiwan

[*] Corresponding author

The corresponding author with contact details

Jiz-Yuh Wang, Ph.D.

Full postal address: Graduate Institute of Medicine, College of Medicine, Kaohsiung Medical University, No.100, Shih-Chuan 1st Road, Sanmin District, Kaohsiung 80708, Taiwan.

Active e-mail address: jizyuhwang@kmu.edu.tw

Supplementary Materials and Methods

Antibodies

Antibodies, including ERK (#4695), JNK (#9252), p38 (#9212), Akt (#4685), NFκB p65 (#8242), IκBα (#9242), p53 (#2524), pERK (#4377), pJNK (#9251), pp38 (#4511), pAkt (#9271), pNFκB p65 (#3033), pIκBα (#2859), pp53 (#9284), p62 (#5114), LC3B (#83506), Bcl-2 (#3498), caspase-3 (#9662), cleaved caspase-3 (#9664), HSP70 (#4872), NLRP3 (#15101), COX-2 (#12282), iNOS (#13120), and lamin B1 (#13435) were purchased from Cell Signaling Technology (Beverly, MA, USA). HO-1 (ADI-SPA-895) and SGLT2 (24654-1-AP) antibodies were purchased from Enzo Life Sciences (Farmingdale, NY, USA) and Proteintech Group (Rosemont, IL, USA), respectively. Antibodies specific for GAPDH (sc-32233) and BrdU (sc-32323) were purchased from Santa Cruz Biotechnology (Santa Cruz, CA, USA). Alexa Fluor 488 (Goat Anti-Mouse: 115-545-003 and Goat Anti-Rabbit: 111-545-003) and horseradish peroxidase (HRP; goat anti-mouse: 115-035-003 and goat anti-rabbit: 111-035-003)-conjugated secondary antibodies were purchased from Jackson ImmunoResearch Laboratories (West Grove, PA, USA).

BrdU Labeling for In Vitro Cell Proliferation Assay

For BrdU immunofluorescence, cells treated for 24 h as indicated were fixed with 4% paraformaldehyde dissolved in PBS for 20 min at room temperature. After permeabilization with 0.2% Triton X-100 for 10 min, the cells were blocked with 5% normal goat serum plus 3% bovine serum albumin for 1 h at room temperature and then washed with 0.2% Triton™ X-100 for 10 min. To denature the DNA, cells were incubated with 2 N HCl for 30 min at room temperature and then subjected to another incubation with 0.1 M sodium borate buffer (pH 8.5) to neutralize the acid. After washing twice with PBS, the cells were incubated with the primary mouse monoclonal BrdU antibody (1 : 200) overnight at 4°C, followed by treatment with Alexa Fluor 488-conjugated goat anti-mouse secondary antibody (1 : 400) applied for 2 h at room temperature, in the dark. For nuclear staining, a popular blue fluorescent DNA dye DAPI was applied. Images were taken with the CCD camera connected to an Axioplan 2 fluorescence microscope (Zeiss, Oberkochen, Germany). BrdU incorporation was quantified by calculating BrdU-positive cells as a percentage of the total number of DAPI-labeled nuclei using the ImageJ software (ImageJ/Fiji v. 1.52p, Http://imagej.nih.gov/ij, USA) for image analysis. At least 20 randomly selected fields for each treatment group were subjected to examination.

Western Blotting and Subcellular Fractionation

The cells were first washed once in cold PBS and lysed in RIPA buffer [50 mM Tris/HCl, pH 8.0, 150 mM NaCl, 1% Nonidet P-40, 0.5% sodium deoxycholate, 0.1% sodium dodecyl sulfate

(SDS)] containing a protease and phosphatase inhibitor cocktail (Sigma-Aldrich). After lysing the cells on ice for 20 min, the total cell lysates were collected by centrifugation at 1.4×10^4 g for 10 min (4°C). Protein concentrations were determined using a BCA kit (Thermo Scientific, Rockford, IL, USA). Thereafter, equal amounts (30 μg) of cell lysate proteins were separated by electrophoresis on an SDS-polyacrylamide gel, and transferred to polyvinylidene fluoride (PVDF) membranes (Millipore, Bedford, MA, USA). The membranes were incubated with primary antibodies, then with anti-mouse or anti-rabbit secondary antibodies conjugated with HRP. Enhanced chemiluminescence method (Pierce; Thermo Fisher Scientific) was used to visualize the proteins bound to the HRP-conjugated antibodies. Then, to quantify the observed protein presentation, the expression level of each western blot band was assessed by densitometric analysis using ImageJ software. Data are expressed as densitometric values relative to the group treated with NG (assigned a value of 1). GAPDH served as the protein-loading control.

In addition, to ensure that all experimental samples and controls used for one comparative analysis are run on the same blot/gel, PVDF membranes were stripped with Restore™ Western Blot Stripping Buffer (Thermo Scientific, Rockford, IL, USA) or subjected to a stripping protocol (100 mM β-mercaptoethanol, 2% SDS, and 62.5 mM Tris pH 6.7 at 50°C for 30 min), and then reprobed with another primary antibodies to determine expression of the protein of interest or with anti-GAPDH antibody to verify equal protein loading between lanes.

For the preparation of nuclear and cytosolic protein fractions, a commercially available kit (K266-25, BioVision, Mountain view, CA) was used according to the manufacturer's instructions.

Supplementary Figures

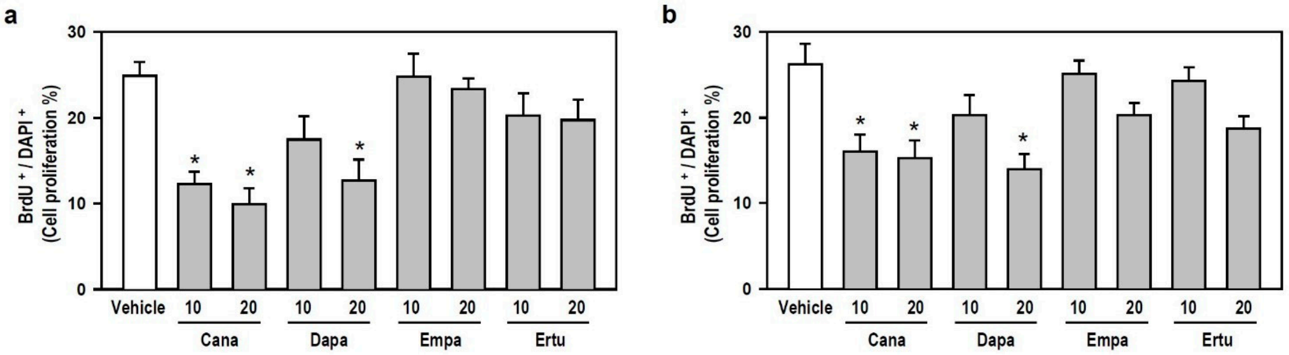

Figure S1. Two additional replicates of the *in vitro* cell proliferation assay conducted using BrdU labeling. The results showed that both Cana and Dapa significantly decreased the proliferation of cultured BV-2 cells. Quantitative results were visualized using histograms, and values are expressed as mean ± SEM. *$p < 0.05$ vs. vehicle-treated control group.

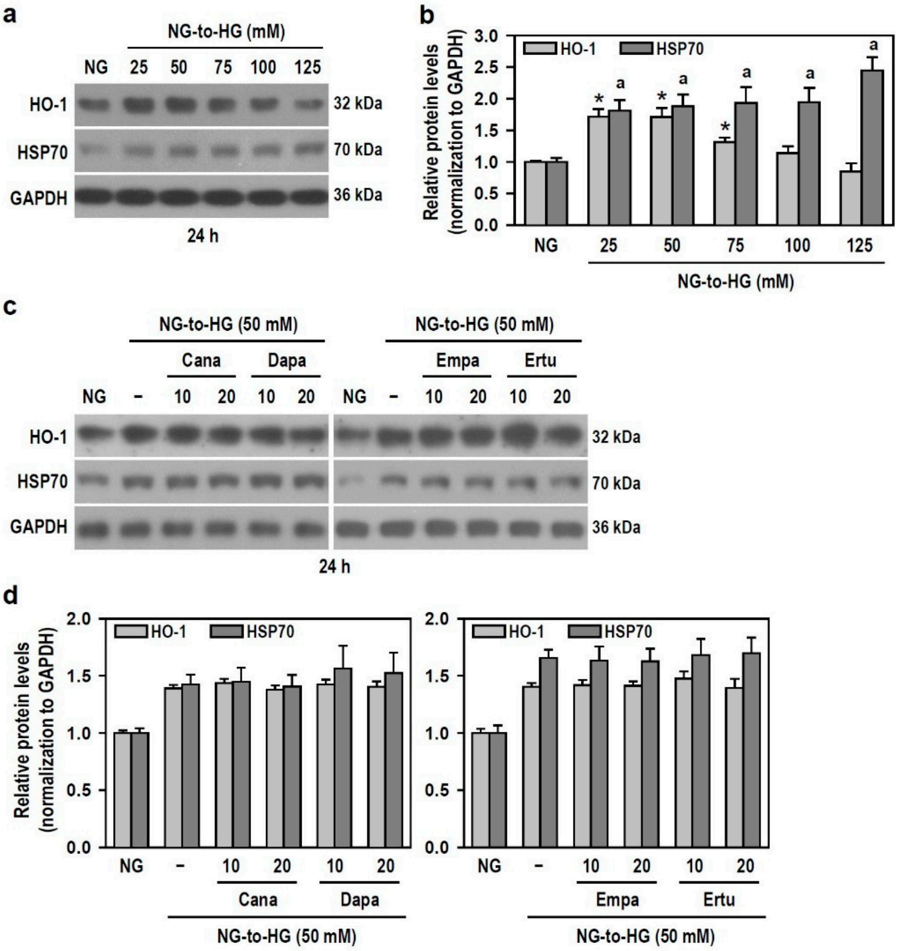

Figure S2. All tested SGLT2 inhibitors show no impact on the HG-induced expression of HO-1 and HSP70 in BV-2 cells. (**a,b**) NG-cultured cells were untreated or treated with a series of HG (25–125 mM) for 24 h; (**c,d**) alternatively, cells were stimulated with 50 mM HG for 24 h, with or without the four SGLT2 inhibitors (10 and 20 μM) given 30 min in advance. NG-treated cultures served as the control group. The levels of HO-1 and HSP70 induction were analyzed using Western blotting. Quantitative results were presented as histograms, with relative densitometric analysis values expressed as mean ± SEM (n = 4). *$p < 0.05$ vs. NG group's HO-1 and $^{a}p < 0.05$ vs. NG group's HSP70.

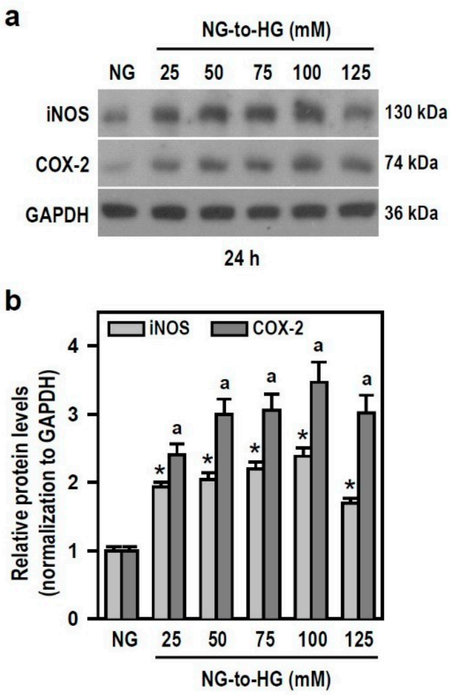

Figure S3. HG induces iNOS and COX-2 expression in BV-2 cells in a concentration-dependent manner. (**a**) NG-cultured cells were exposed to a series of HG treatments (25–125 mM) for 24 h, while untreated NG-cultured cells were used as the control group. The levels of iNOS and COX-2 induction were analyzed using Western blotting. (**b**) Quantitative results were presented as histograms, with relative densitometric analysis values expressed as mean ± SEM (n = 4). *$p < 0.05$ vs. NG group's iNOS; $^{a}p < 0.05$ vs. NG group's COX-2.

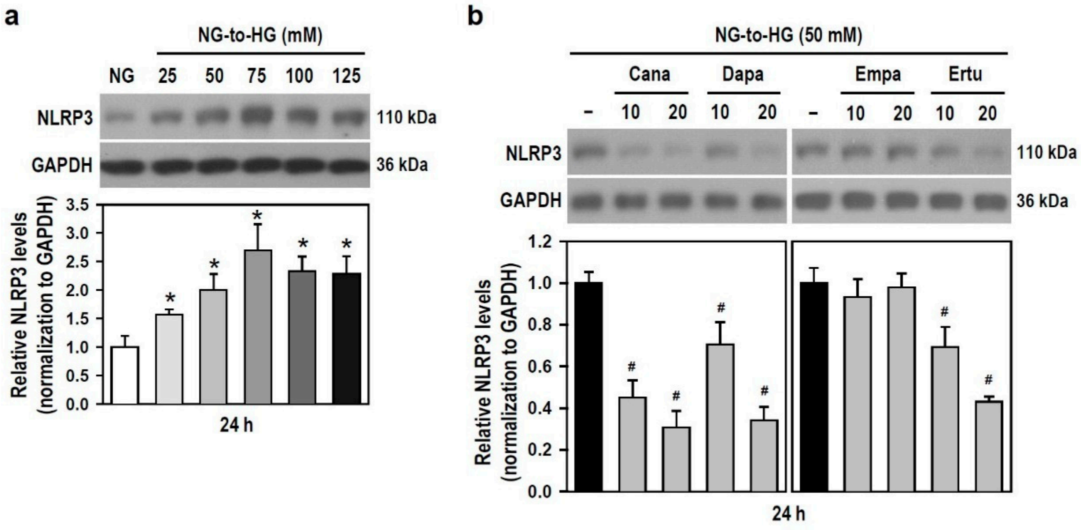

Figure S4. HG stimulation leads to an upregulation of NLRP3 expression, which is effectively inhibited by Cana, Dapa, and Ertu, but not Empa. (**a**) NG-cultured cells were untreated or treated with a series of HG (25–125 mM) for 24 h. (**b**) Cultures, with or without the four SGLT2 inhibitors (10 and 20 μM) added 30 min in advance, were subjected to 50 mM HG treatment for 24 h. The expression levels of NLRP3 were analyzed using Western blotting. Quantitative results were presented in histograms, with densitometric analysis values expressed as mean ± SEM (n = 4). $^{*}p < 0.05$ vs. NG group and $^{\#}p < 0.05$ vs. HG group.

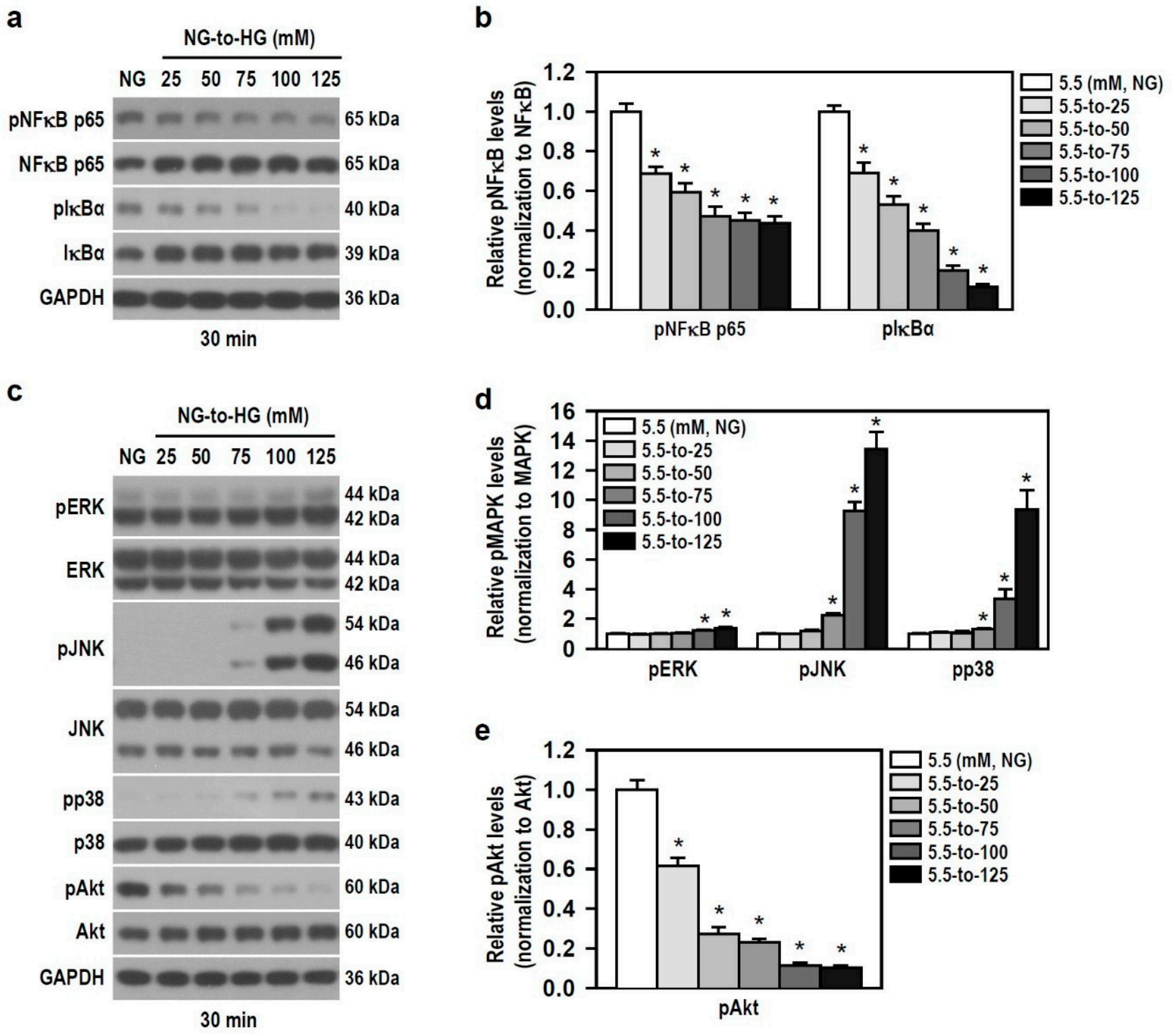

Figure S5. HG concentration-dependently alters the activity of NFκB, MAPKs, and Akt in BV-2 cells. NG-cultured cells were subjected to a range of HG concentrations (25–125 mM) for 30 min, with NG-treated cultures serving as the control group. Protein levels were analyzed using Western blotting. (**a,b**) The levels of pNFκB p65, NFκB p65, pIκBα, and IκBα were detected; (**c**–**e**) alternatively, the levels of pERK, ERK, pJNK, JNK, pp38, p38, pAkt, and Akt were determined. The blot collection in each individual panel was obtained from the same membrane that underwent stripping/reprobing. Densitometric analysis results were presented in histograms and are expressed as mean ± SEM (n = 4). *$p < 0.05$ vs. NG group.

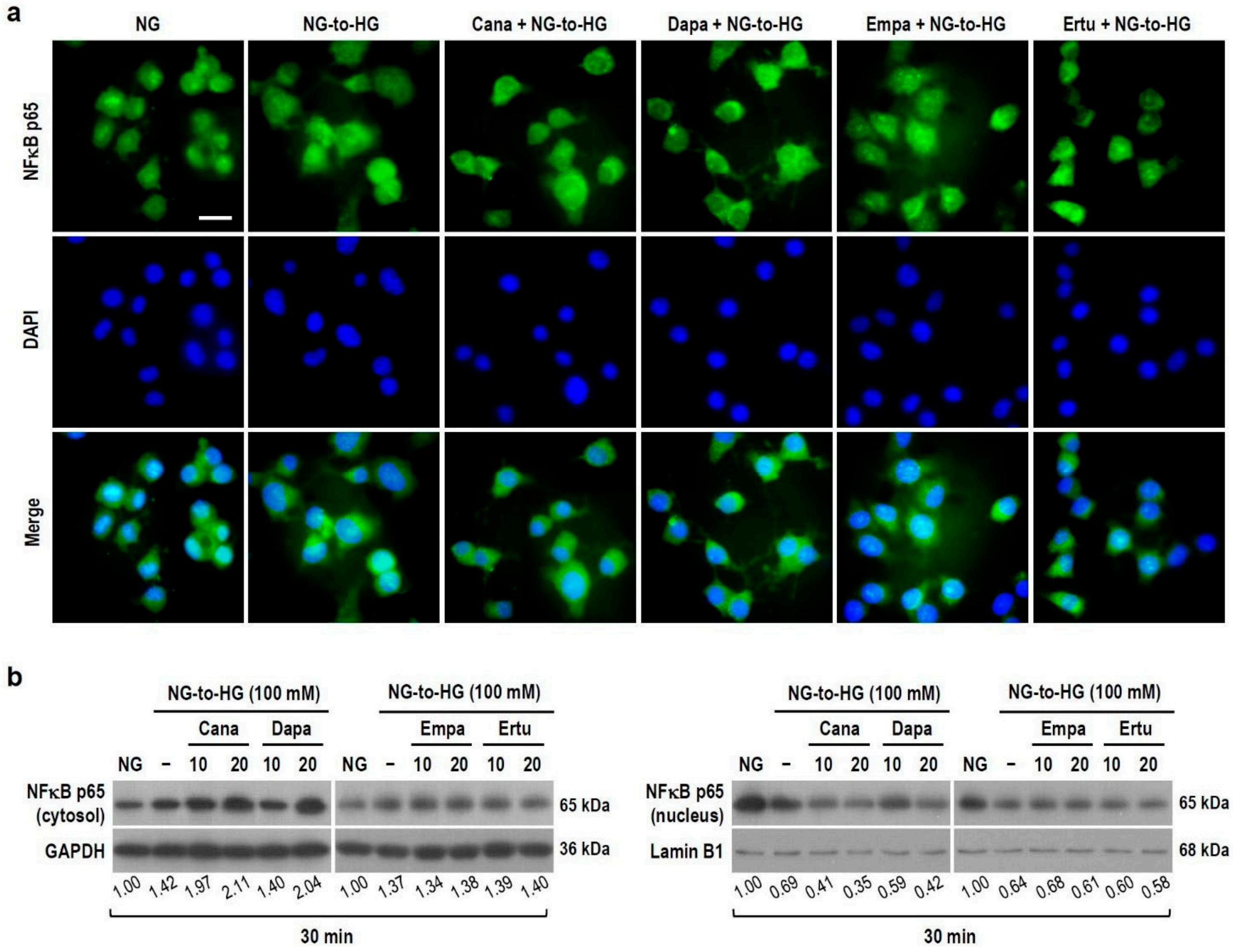

Figure S6. Both Cana and Dapa further increase the translocation of HG-induced NFκB p65 to the cytoplasm of BV-2 cells. (**a**) Cells cultured under NG conditions were treated with 100 mM HG for 30 min, with or without the addition of the four SGLT2 inhibitors (20 μM) 30 minutes prior. The translocation of NFκB p65 was assessed using immunofluorescence staining with an NFκB p65 antibody and an Alexa Fluor 488-conjugated antibody. Nuclei were counterstained with DAPI. Scale bar = 25 μm. (**b**) The same treatments as in (**a**) were administered, and the four SGLT2 inhibitors were applied at two concentrations (10 and 20 μM). NG-treated cultures were used as the control group. Nuclear and cytosolic proteins were extracted using a commercial Nuclear/Cytosol Fractionation Kit. The levels of NFκB p65 translocation were analyzed by Western blotting. After normalization to either GAPDH (a cytosolic marker protein) or lamin B1 (a nuclear marker protein), the densitometric analysis values of NFκB p65 from a single independent experiment were presented below the representative blots.

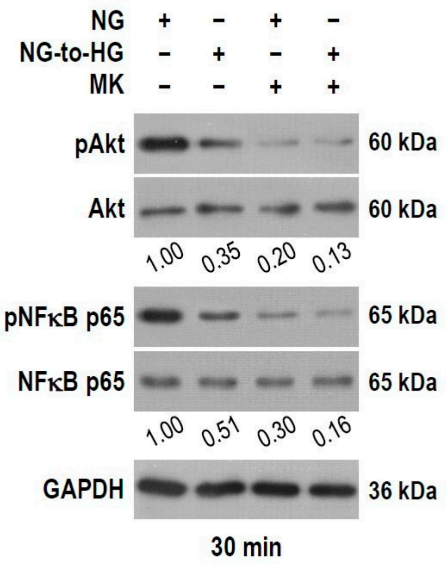

Figure S7. The phosphorylated NFκB p65 level in BV-2 cells is reduced by the Akt inhibitor MK2206. NG-cultured cells were treated with HG (100 mM) or MK2206 (MK; 10 μM) alone for 30 min. For the co-treatment of MK2206 and HG, MK2206 was added 30 min prior to the administration of HG and was continuously present along with HG for 30 min. NG-treated cultures were used as the control group. The levels of pAkt, Akt, pNFκB p65, and NFκB p65 were analyzed using Western blotting. Densitometric analysis values obtained from a single independent experiment were displayed below the representative blots.

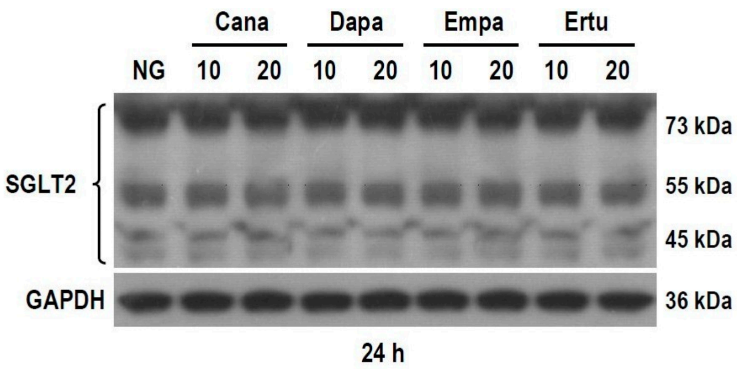

Figure S8. SGLT2 inhibitors fail to induce any changes in the levels of SGLT2 protein in NG-cultured BV-2 cells. Cells cultured under NG conditions were treated with or without the four SGLT2 inhibitors (10 and 20 μM) for 24 h. The untreated cultures served as the control group. Western blotting was used to analyze SGLT2 protein levels. The representative blots shown were from one of three independent experiments.

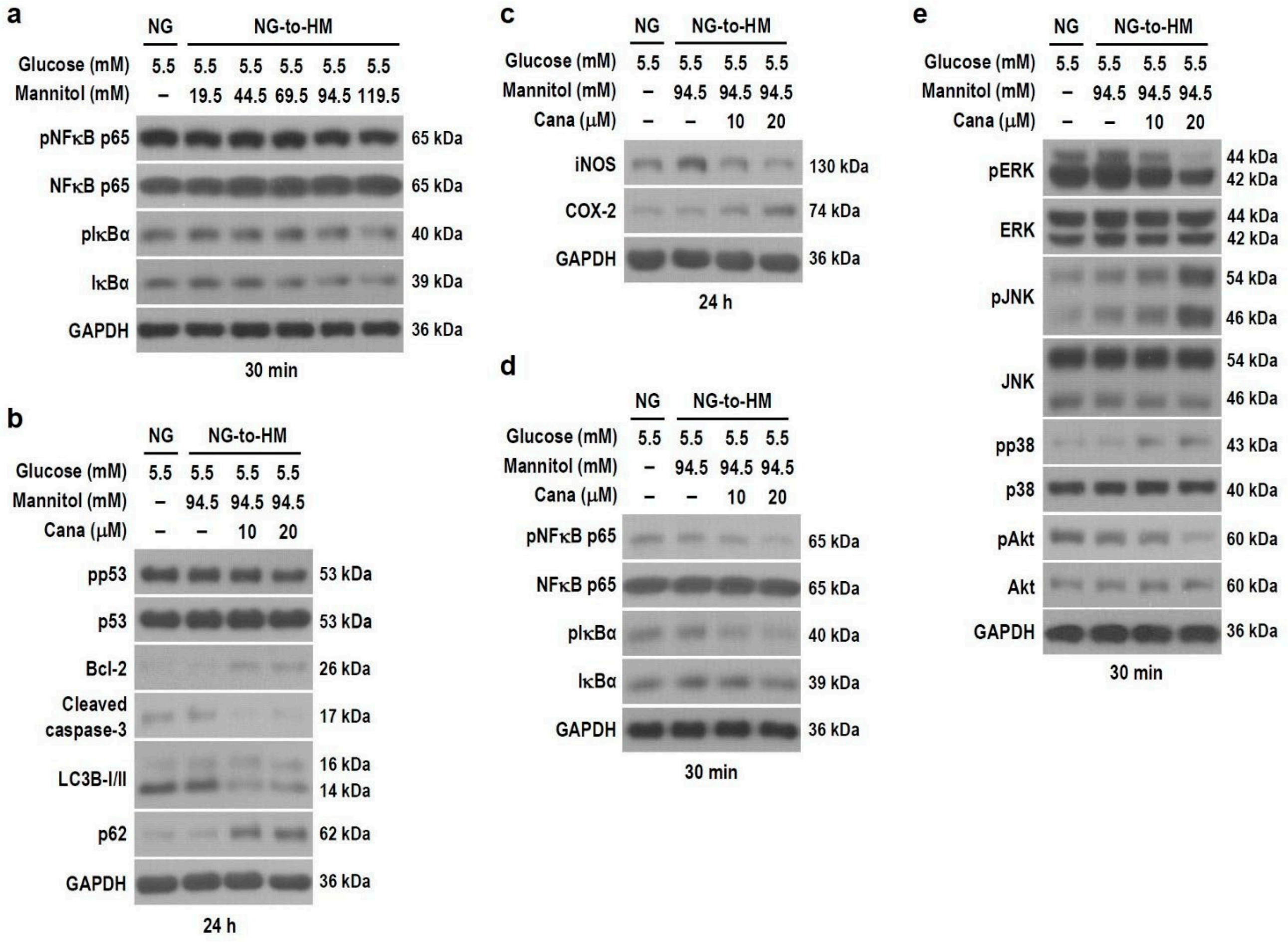

Figure S9. Although high mannitol triggers immune inflammation not by altering NFκB activity and fails to elicit significant cellular lethality, Cana still shows anti-inflammatory efficacy in BV-2 cells by modulating the activities of NFκB, MAPKs, and Akt. (**a**) NG-cultured cells were subjected to a range of high mannitol (HM) concentrations (19.5–119.5 mM) for a duration of 30 min. The levels of pNFκB p65, NFκB p65, pIκBα, and IκBα were determined. (**b,c**) Cells cultured under NG conditions were treated with 94.5 mM HM for 24 h, without the addition of SGLT2 inhibitor Cana or with Cana (10 and 20 μM) added 30 min in advance. The levels of apoptotic, autophagic, and inflammatory markers, as indicated, were evaluated. (**d,e**) The same treatment as in (**b, c**) was performed, but the cultures were treated for a duration of 30 min. The levels of inflammation-related signaling kinases and transcription factors, as indicated in the Figure, were assessed. GAPDH was utilized as a loading control for protein analysis. Cultures treated with NG displaying normal osmolarity served as the control group. Western blotting was performed to analyze the expression levels of the proteins of interest. The blot collection in each individual panel was obtained from the same membrane that underwent stripping/reprobing. The representative blots shown were from one of three independent experiments.

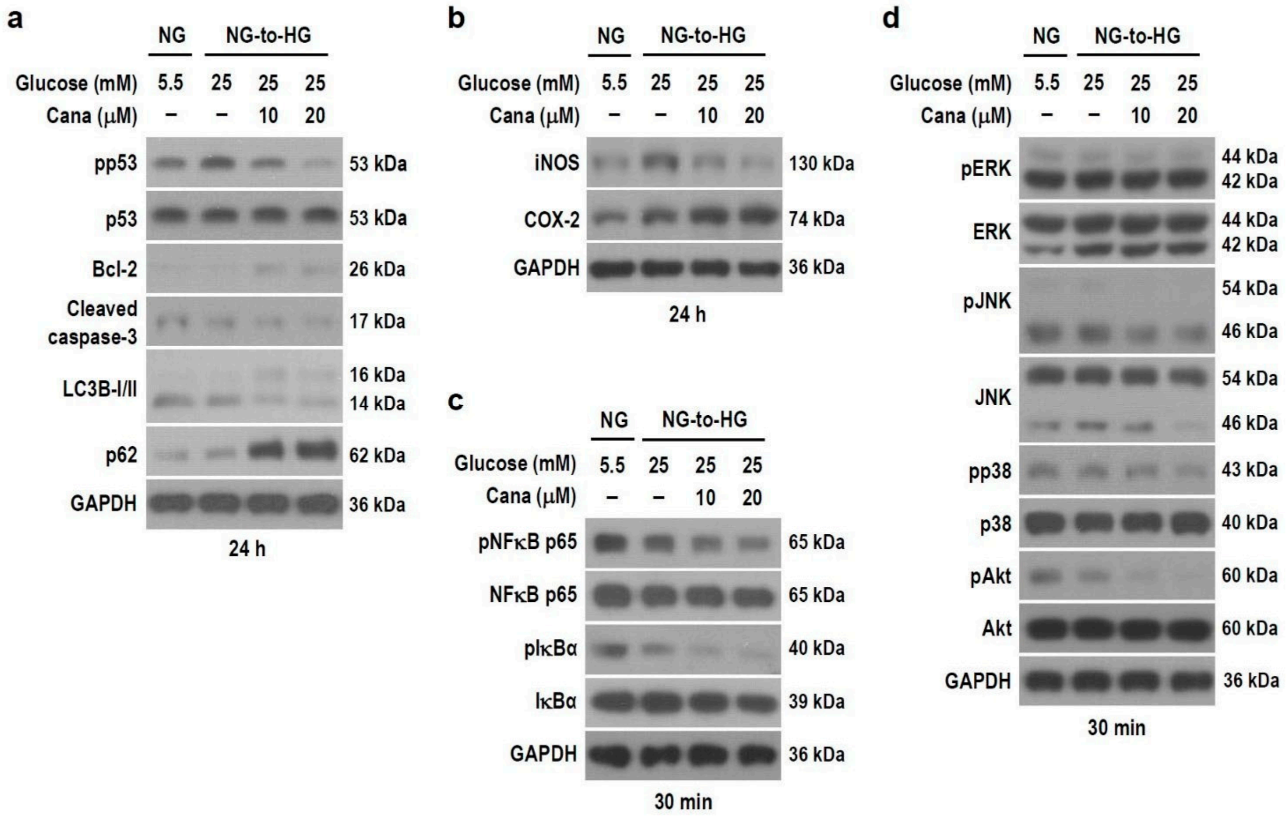

Figure S10. Cana alleviates immune inflammation and associated signaling cascades induced by 25 mM HG in BV-2 cells, even despite the lack of substantial cell death/damage caused by apoptosis and autophagic degradation. (**a,b**) Cells cultured under NG conditions were treated with HG (25 mM) for 24 h, without the addition of SGLT2 inhibitor Cana or with Cana (10 and 20 μM) added 30 min in advance. The levels of apoptotic, autophagic, and inflammatory markers, including pp53, p53, Bcl-2, cleaved caspase-3, LC-3B, p62, iNOS, and COX-2, were evaluated. (**c,d**) The same treatment as in (**a,b**) was performed, but the cultures were treated for a duration of 30 min. The levels of inflammation-related signaling kinases and transcription factors, including pNFκB p65, NFκB p65, pIκBα, IκBα, pERK, ERK, pJNK, JNK, pp38, p38, pAkt, and Akt, were determined. GAPDH was used as a loading control for protein analysis. NG-treated cultures were used as the control group. Western blotting was performed to analyze the expression levels of the proteins of interest. The blot collection in each individual panel was obtained from the same membrane that underwent stripping/reprobing. The representative blots shown were from one of three independent experiments.

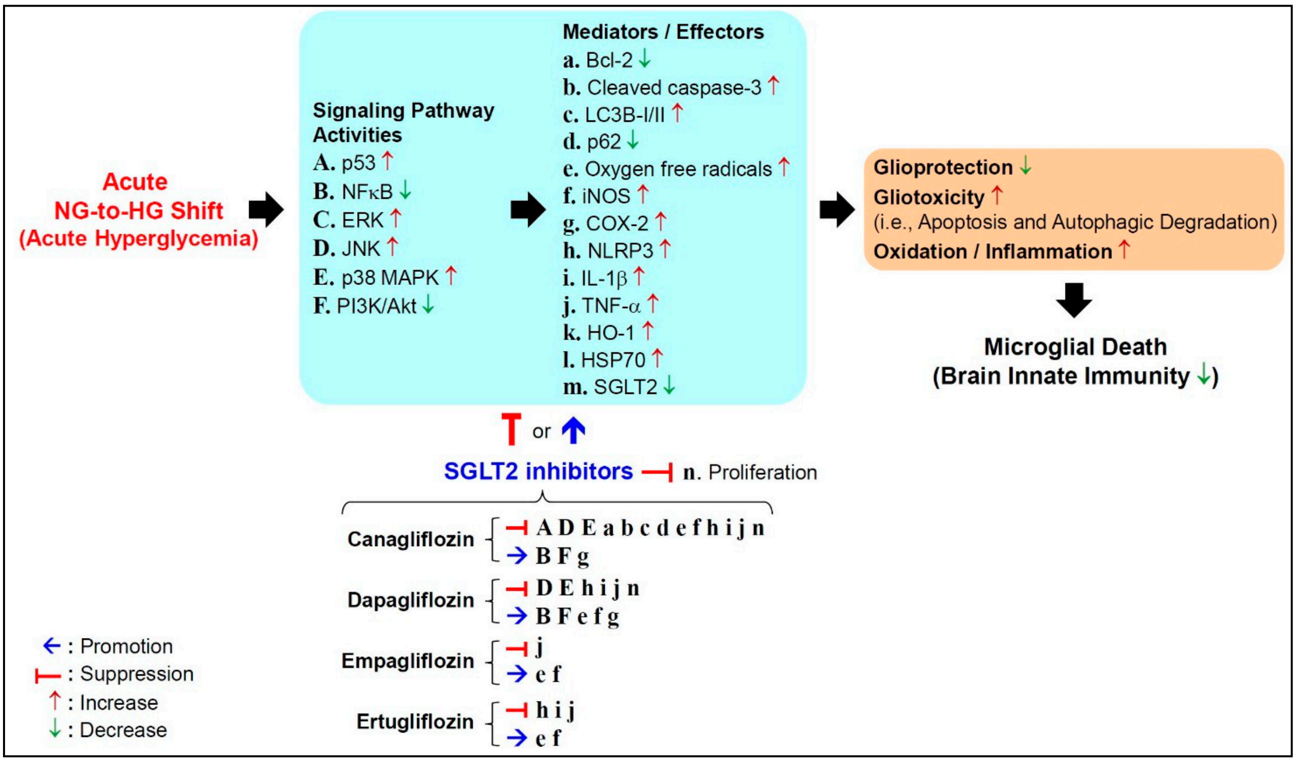

Figure S11. A summary illustration is provided to demonstrate how four SGLT2 inhibitors protect BV-2 microglia from HG-induced inflammatory toxicity. Among the tested SGLT2 inhibitors, Cana exhibited the most significant efficacy in reducing the overall inflammatory stress induced by acute NG-to-HG fluctuation, which mimics an acute hyperglycemic condition.
